# Decoding and steering spatially resolved cellular dynamics

**DOI:** 10.1038/s44320-026-00216-7

**Published:** 2026-05-15

**Authors:** Jiahao Wu, Suoqin Jin

**Affiliations:** School of Mathematics and Statistics, Wuhan University, Wuhan, China

**Keywords:** Computational Biology

## Abstract

This News &Views highlights a recent study by Raghavan and colleagues (in this issue of MSB) that introduces veloAgent, a computational framework that integrates transcriptional dynamics with spatial information to dissect spatially resolved RNA velocity.

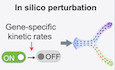

## Teaser

Understanding how cells transition between states is central to biology. RNA velocity has emerged as a transformative framework for inferring future cellular states from unspliced and spliced mRNA transcripts. A new deep generative and agent‑based method can decode and manipulate cellular dynamics within native tissue by dissecting spatially resolved RNA velocity and simulating regulatory interventions, opening new opportunities to explore how tissue context shapes and potentially controls cell‑state transitions.

**Figure 1:**
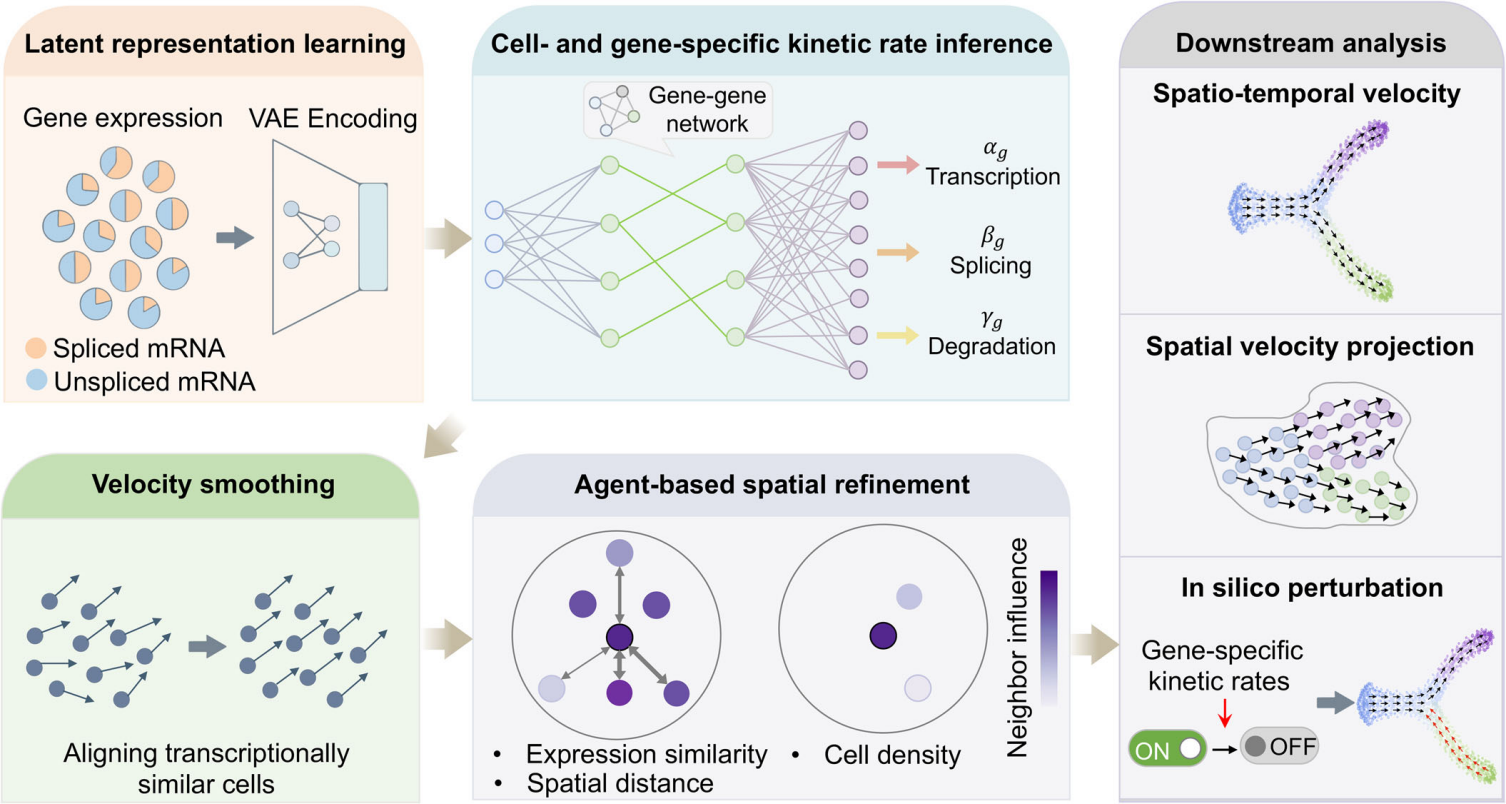
Overview of the veloAgent framework and downstream analysis. The veloAgent framework takes spliced and unspliced mRNA as input and encodes them into a low-dimensional latent representation using a variational autoencoder (VAE). The latent representation is then fed into a neural network constructed in conjunction with gene–gene interaction networks to predict cell- and gene-specific kinetic parameters, including transcription (α), splicing (β), and degradation (γ) rates, from which initial RNA velocity is computed. The inferred RNA velocity is subsequently refined in two steps. First, velocity smoothing is performed to enforce local coherence by aligning the directions of transcriptionally similar cells. Second, an agent-based model (ABM) is applied for spatial refinement, and its velocity is adjusted through neighborhood-based weighting that incorporates spatial distance, local cell density, and expression similarity. Finally, the refined RNA velocity is used for downstream analysis, including spatio-temporal dynamics inference, projection of velocity fields onto low-dimensional space and spatial tissue map, and in silico gene perturbation of cellular dynamics.

Understanding how cells transition between states is central to biology, from development to disease progression. Although single-cell RNA sequencing (scRNA-seq) provides high-resolution snapshots of cellular states, it does not directly capture the dynamic processes underlying cell state transitions. RNA velocity represents a major advance in this context, as it enables the inference of cellular state transitions by leveraging the abundance of unspliced and spliced RNA, providing a dynamic view of gene expression and allowing the extrapolation of future cellular states (Bergen et al, 2021). This concept was first introduced by La Manno et al (2018), and later extended by relaxing the steady-state assumption and improving the estimation of transcriptional kinetics (Bergen et al, 2020; Li et al, 2024). Despite these advances, existing RNA velocity methods often produce inferred cellular dynamics that are inconsistent with tissue organization, largely because they do not explicitly account for spatial context. Advances in spatial transcriptomics (ST) now enable the measurement of gene expression together with spatial location, offering an opportunity to study how cellular dynamics unfold within tissues (Marx, 2021). In a recent article published in *Molecular Systems Biology*, Raghavan et al (2026) introduce veloAgent, a computational framework that integrates transcriptional dynamics with spatial information to dissect spatially resolved RNA velocity and steer cellular dynamics within native tissue (Fig. 1).

Instead of using raw spliced and unspliced counts directly for modeling transcriptional dynamics, veloAgent encodes these expression data into a latent representation per cell using a variational autoencoder (VAE), reducing dimensionality while capturing a robust transcriptional state. Its key advance is an agent‑based formulation: cells are modeled as interacting entities within their local microenvironment. Rather than treating cells independently or using spatial information as an auxiliary feature, veloAgent directly leverages spatial context to constrain and guide the inference of cellular dynamics, allowing transitions to emerge from local neighbor interactions. This shift from independent observations to interacting agents provides a more realistic representation of how cellular processes unfold in tissues. Moreover, integration of gene–gene interaction structures introduces biologically grounded constraints that link transcriptional dynamics to regulatory mechanisms, forming the basis for in silico perturbation analysis.

veloAgent incorporates a mechanistic RNA velocity model parameterized by gene-specific kinetic rates, including transcription (α) and splicing (β). In this framework, in silico perturbation is implemented by directly modulating these parameters for individual genes—such as altering α to simulate transcriptional activation or repression—and subsequently recomputing the induced velocity field. This formulation ensures that perturbations are applied at the level of the underlying dynamical system, rather than to observed expression values. The functional impact of each perturbation is quantified by evaluating changes in the directionality of cellular velocity vectors, particularly with respect to predefined target states or trajectories. Genes are prioritized based on their ability to reorient cellular dynamics toward these targets, thereby identifying candidate regulators of cell state transitions. Because perturbations are performed within the learned parameter space, this approach enables efficient, large-scale screening without retraining, while maintaining mechanistic interpretability.

veloAgent has been successfully applied to diverse ST datasets from multiple platforms. In structured tissues such as the brain, veloAgent produced trajectories that closely aligned with known anatomical organization, avoiding the inconsistent or anatomically implausible directions observed in earlier approaches. In silico perturbation analysis identified key regulatory genes whose modulation led to a clear reversal of progression toward terminal fiber-tract cell states. In a breast cancer dataset, the inferred transitions were more consistent with expected tumor progression patterns, correcting misdirected flows produced by competing methods. Further perturbation analysis revealed potential therapeutic targets that suppress malignant progression. Quantitatively, veloAgent outperforms other methods in cross‑boundary directionality, fate probability, and velocity confidence. Notably, veloAgent also improves temporal velocity estimation for scRNA‑seq data even in the absence of spatial information. These findings underscore the importance of studying cellular dynamics within native environments. Cellular behavior is shaped not only by transcriptional states but also by spatial structure, physical constraints, and local interactions. Importantly, veloAgent provides a mechanistically grounded framework for simulating gene perturbations, enabling the identification of key regulatory genes and potential therapeutic targets.

Despite the advances of veloAgent, several promising future directions remain. First, ST data are often collected across multiple tissue sections and conditions, posing challenges for batch correction and robust cross‑sample comparison of cellular dynamics. Second, integrating additional modalities (e.g., epigenomics, proteomics) would provide a more comprehensive view of regulatory processes underlying cell dynamics (Vandereyken et al, 2023). Third, while veloAgent provides a foundation for predictive intervention models, incorporating causal machine learning frameworks (Tejada-Lapuerta et al, 2025) could further enhance interpretability and predictive power. Fourth, veloAgent approximates neighborhood effects under spatial constraints rather than explicitly modeling intercellular signaling; given that cellular behavior is strongly shaped by cell–cell communication (Jin et al, 2025), directly modeling such interactions would deepen mechanistic understanding. Looking ahead, veloAgent opens new opportunities to explore how tissue context shapes and potentially controls cell‑state transitions. An important direction is to move beyond descriptive analysis toward predictive models capable of in silico perturbation of cellular behavior (Bunne et al, 2024), which would accelerate therapeutic target discovery and advance precision medicine.
